# Floating Ice-Algal Aggregates below Melting Arctic Sea Ice

**DOI:** 10.1371/journal.pone.0076599

**Published:** 2013-10-16

**Authors:** Philipp Assmy, Jens K. Ehn, Mar Fernández-Méndez, Haakon Hop, Christian Katlein, Arild Sundfjord, Katrin Bluhm, Malin Daase, Anja Engel, Agneta Fransson, Mats A. Granskog, Stephen R. Hudson, Svein Kristiansen, Marcel Nicolaus, Ilka Peeken, Angelika H. H. Renner, Gunnar Spreen, Agnieszka Tatarek, Jozef Wiktor

**Affiliations:** 1 Norwegian Polar Institute, Fram Centre, Tromsø, Norway; 2 University of Manitoba, Centre for Earth Observation Science, Winnipeg, Canada; 3 Alfred-Wegener-Institut Helmholtz-Zentrum fùr Polar- und Meeresforschung, Bremerhaven, Germany; 4 Max Planck Institute for Marine Microbiology, Bremen, Germany; 5 Akvaplan-niva, Fram Centre, Tromsø, Norway; 6 GEOMAR Helmholtz Centre for Ocean Research Kiel, Kiel, Germany; 7 Department of Arctic and Marine Biology, University of Tromsø, Tromsø, Norway; 8 MARUM - Center for Marine Environmental Sciences, University of Bremen, Bremen, Germany; 9 Institute of Oceanology, Polish Academy of Science, Sopot, Poland; University of Shiga Prefecture, Japan

## Abstract

During two consecutive cruises to the Eastern Central Arctic in late summer 2012, we observed floating algal aggregates in the melt-water layer below and between melting ice floes of first-year pack ice. The macroscopic (1-15 cm in diameter) aggregates had a mucous consistency and were dominated by typical ice-associated pennate diatoms embedded within the mucous matrix. Aggregates maintained buoyancy and accumulated just above a strong pycnocline that separated meltwater and seawater layers. We were able, for the first time, to obtain quantitative abundance and biomass estimates of these aggregates. Although their biomass and production on a square metre basis was small compared to ice-algal blooms, the floating ice-algal aggregates supported high levels of biological activity on the scale of the individual aggregate. In addition they constituted a food source for the ice-associated fauna as revealed by pigments indicative of zooplankton grazing, high abundance of naked ciliates, and ice amphipods associated with them. During the Arctic melt season, these floating aggregates likely play an important ecological role in an otherwise impoverished near-surface sea ice environment. Our findings provide important observations and measurements of a unique aggregate-based habitat during the 2012 record sea ice minimum year.

## Introduction

The ongoing thinning and loss of Arctic sea ice will lead to changes in the surface energy budget of the Arctic Ocean [1,2] and will have far-reaching ramifications for both sympagic (ice-associated) and pelagic ecosystems [3]. Nonetheless, thorough documentation of effects on Arctic marine biota in response to climate change is limited, especially for planktonic and ice-associated ecosystems [4]. Some knowledge gaps are attributable to the limited amount of quantitative data on production, consumption and biomass for Arctic marine ecosystems [5]. A recent study revealed significant changes in planktonic microbial community structure before and after the September 2007 record sea ice minimum [6]. Achieving greater understanding of these processes is important because the increased and earlier loss of Arctic sea ice will not only affect the timing of ice algal and phytoplankton blooms [7], but could also lead to trophic mismatch scenarios between primary producers and their dependant grazers [8].

Melting of summer sea ice results in habitat deterioration for ice-associated organisms and the formation of a stratified surface meltwater layer [9,10]. Ice algae, at the base of the Arctic sea ice ecosystem, have to cope with being released into freshening surface water during the melt season. Due to their inherent stickiness, they are prone to aggregation [11] and subsequent sedimentation [12]. Vertical flux and sedimentation of ice-algal material will transfer energy to pelagic and benthic ecosystems [13-16] and deprive ice-associated fauna of their food resource [9]. In order to bridge the gap between release from melting sea ice in summer and reincorporation into the ice at the onset of freezing during autumn, ice algae must rely on other means to stay in close proximity to the sea-ice habitat, especially over the deep Central Arctic Basin. A recent study [17] suggests that progressively thinning Arctic sea ice can also lead to new ice-associated habitats. The authors propose that incorporation of algal cells in the soft ice of open melt ponds could account for the algal masses they observed at the surface of refrozen melt ponds in early autumn. Buoyant, free-floating ice-algal aggregates have previously been observed in the Arctic [18-21], but due to their patchy distribution little is known about their ecological significance during the melt season. Moreover, their potential role in seeding the next spring bloom has not been considered thus far. In addition, current models of ice algal primary production and biomass do not account for these aggregations or the metre-long mats and strands formed by the under-ice diatom *Melosira arctica* [20], and therefore underestimate the contribution of ice algae to total primary production in the Arctic Ocean [22].

Large numbers of ice-algal aggregates were encountered during two consecutive research cruises to the Eastern Central Arctic Ocean in summer 2012: (1) the Centre for Ice, Climate and Ecosystems (ICE) cruise with RV *Lance* (ICE12) and (2) IceArc expedition ARK-XXVII/3 with RV *Polarstern*. In order to elucidate the significance of these aggregates, we sampled and quantified their abundance, biomass and production using three different approaches: (i) trapping of aggregates in an ice hole (“inverted sediment trap” approach), (ii) collection by scuba divers, and (iii) under-ice video transects with a remotely operated vehicle (ROV). Combined, these different approaches enabled us to extrapolate our findings to a square metre scale and assess the significance of the floating ice-algal aggregates for the ice-associated ecosystem during the oligotrophic summer months.

## Materials and Methods

### Study area

**Figure 1:**
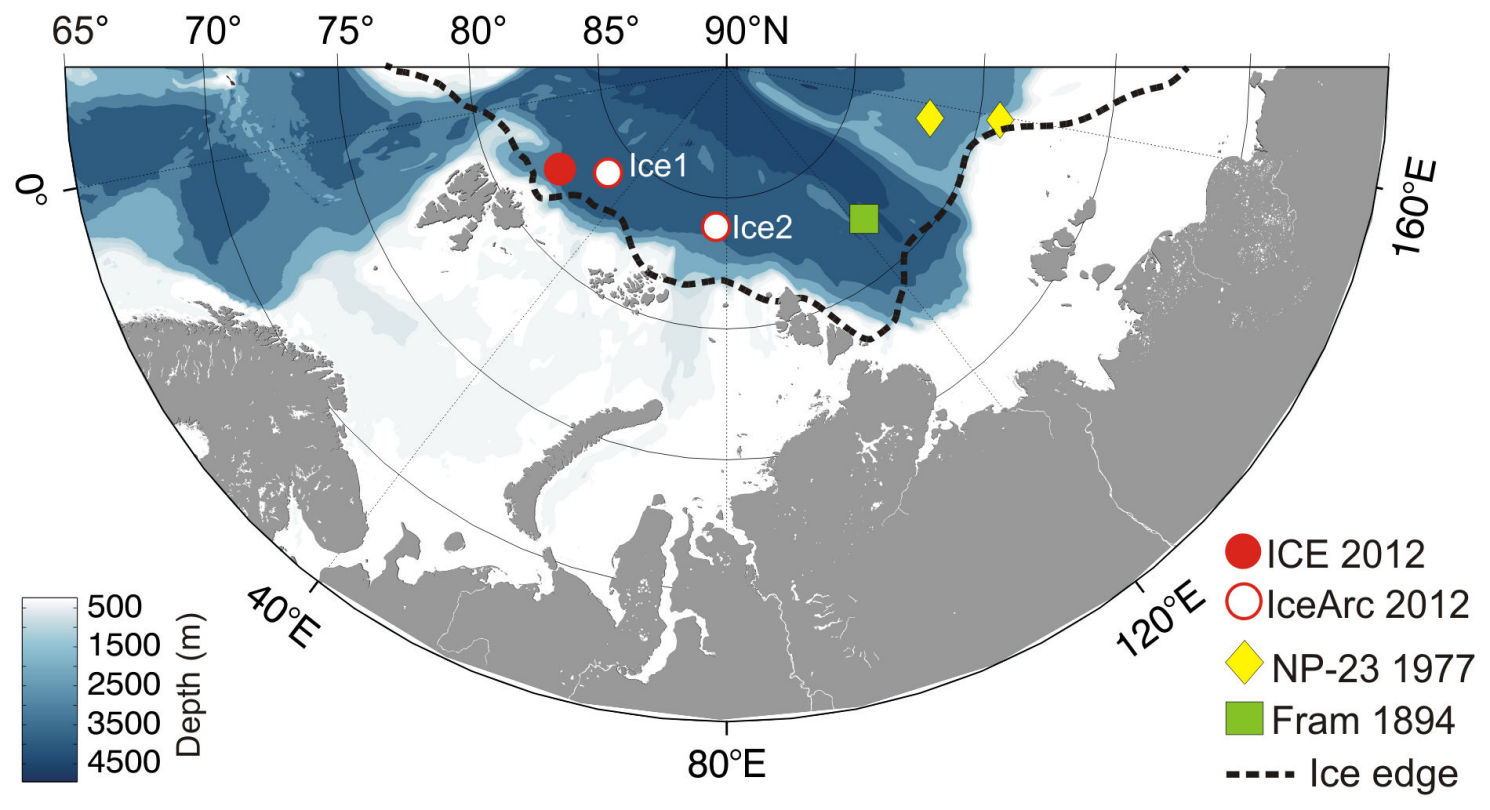
Ice-algal aggregate observations recorded in the Eastern Central Arctic during the Centre for Ice, Climate and Ecosystems (ICE12) cruise with RV *Lance*, IceArc expedition ARK-XXVII/3 with RV *Polarstern*, the Russian North Pole drift station NP-23 and the Fram Expedition 1893-1896. The broken line and blue colour scale indicate the approximate position of the ice edge at the end of July 2012 and the sea floor depth, respectively.

The three drift ice stations were situated over the deep Arctic basin and well north of the ice edge (Figure 1). The pack ice in the area was dominated by first year ice (FYI) and featured well-developed melt ponds. The first long drift ice station was occupied from 26 July to 3 August 2012 during the ICE12 cruise and initially situated at 82.5° N, 21° E north of Svalbard while the two shorter drift ice stations, centred at 84° N, 31° E (station Ice1) and 84° N, 78° E (station Ice2) on 9–11 and 14–16 August 2012 respectively, were undertaken during the subsequent IceArc expedition.

### ICE12

#### Sample collection and preparation

Throughout the drift ice station from 26 July to 3 August, conductivity, temperature and depth (CTD) profiles were taken daily using a rosette system (Seabird Electronics SBE911) deployed from the side of the ship. Seawater samples were collected on 26 and 30 July and daily from 1 to 3 August at five depths (surface, 10, 50, 100 m, Chl *a* maximum). Profiles of salinity, temperature, and dissolved oxygen from the under-ice water column were obtained on 28, 30 and 31 July and 1 August using a hand-held MicroCat sensor (Seabird Electronics 37-SM) lowered through an ice hole. Subsequently, water samples from the meltwater layer underneath the sea ice were pumped through the same ice hole. Additionally video profiles (GoPro Hero2) provided information on the distribution of floating aggregates in cracks and openings in the sea ice. Three video profiles were obtained: one on 29 July at 19:30 GMT and two on 31 July at 08:30 -09:00 GMT through the ice hole used to collect the aggregates (further information below). On 29 July at 13:00 GMT, an additional video profile was obtained through an ice crevice in close proximity to the ice hole. Biological sea ice samples were collected on 28 July and 1 August using an ice corer with an inner diameter of 9 cm (Mark II coring system, KOVACS Enterprises, Inc., USA). Immediately after collection, ice cores were cut into 10-20 cm thick sections with a stainless steel saw, transferred into polyethylene zip-lock bags, stored dark and melted on board the ship at 4°C. Sea ice samples were melted directly, not in filtered seawater, because nutrient samples were taken from the same core sections. Comparison between direct melting and melting in filtered seawater at 4°C showed no significant differences in taxonomic composition of sea ice assemblages [23]. Seawater and meltwater samples as well as melted sea ice samples to be analysed for chlorophyll (Chl *a*), particulate organic carbon (POC) and nitrogen (PON) were filtered onto 25 mm Ø GF/F and pre-combusted (500°C for 4 h) GF/F filters, respectively. Between 500 and 1000 mL of seawater, and meltwater and between 150 and 500 mL of melted sea ice, were sampled for Chl *a*, POC and PON. One replicate sample per depth was collected for each parameter for seawater and meltwater, while sea ice samples were obtained from triplicate ice cores collected within 10 cm of each other. Fifty mL were sampled per analysis of ammonium. On 26 and 29 July, aggregates were collected for species identification with a coarse-meshed sieve from the side of open melt ponds and through a specially drilled ice hole also used to collect accumulated aggregates (further details below). Aggregate samples used for iodine, Chl *a*, and transparent exopolymer particle (TEP) analysis were collected directly underneath the sea ice on 31 July by divers using a slurp gun (modified 3.5 L Trident^®^ suction gun).

#### Chemical and biological measurements

Ammonium was measured on fresh samples directly on board according to Solóranzo [24]. Aggregate samples for iodine speciation were filtered over polycarbonate filters (2 µm pore size) and the filtrate stored frozen (-20°C) until analysis. Iodide was determined by cathodic stripping square wave voltammetry according to the methods of Campos [25] and Luther et al. [26], with a detection limit of 0.1 to 0.2 nmol L^-1^ and a precision better than 5%. High iodide concentrations can be used as a measure of phytoplankton senescence [27].

Eight subsamples (0.5 mL each) of a diver-collected aggregate with a total volume of 38 mL were taken for Chl *a* analysis, after having removed all supernatant seawater. Chl *a* was extracted with 100% methanol at 5°C for 12 h [28] and measured fluorometrically using a Turner Fluorometer 10-AU (Turner Design, Inc.). Phaeopigments were measured after acidification with two drops of 5% HCl. Samples of POC and PON were exposed overnight to 32% HCl prior to analysis in order to remove all particulate inorganic carbon, and were thereafter folded into tin capsules. The carbon content was determined using a CHN elemental analyzer (Euro EA 3000).

For TEP analysis, two diver-collected aggregates were transferred into 15 mL centrifuge tubes and immediately frozen at -20°C. After thawing, the samples were each suspended in 169 mL of <0.2 µm filtered ISOTON (Beckmann Coulter) and divided into subsamples. Subsamples of 3-5 mL, with 4 replicates each, were filtered onto 25 mm polycarbonate filters (Nucleopore 0.4 µm pore size) and stained with Alcian Blue. TEP was measured with the colorimetric method according to Engel [29]. TEP concentrations (given in Gum Xanthan equivalents [Xeq.] L^-1^) were converted to carbon (TEP-C, µg C L^-1^) using a conversion factor of 0.63 [30]. In order to normalize TEP-C to bulk aggregate carbon, the POC concentration of the subsamples was also determined. POC filters were analyzed with an elemental analyser (EuroVektor EA). Measurements of TEP subsamples from samples one and two had a precision of 5 and 15 %, respectively, while measurements of POC subsamples varied by 11%.

For species identification and determination of assemblage composition, aggregates were transferred into brown glass bottles, preserved with a buffered aldehyde mixture (glutaraldehyde: 0.1% final concentration and hexamethylenetetramine-buffered formaldehyde: 1% final concentration) as suggested by Tsuji and Yanagita [31] and stored cool (5°C) and dark for subsequent counting in the home laboratory. An aggregate volume of 0.1–0.2 mL was settled in sedimentation chambers for 24 h. Cells were identified and enumerated using a Nikon Ti-U inverted light and epifluorescence microscope according to the method of Throndsen [32] and counted at 60× magnification. Each sample was examined until at least 500 cells had been counted.

#### Radiation measurements

Ramses-ACC-VIS hyperspectral radiometers (TriOS Mess- und Datentechnik GmbH) were used to measure incoming energy fluxes at the ice surface and transmitted fluxes at the underside of the ice. One instrument was mounted 1.5 m above the ice surface, levelled and facing upwards, while a second was swum by a diver with semi-closed rebreather (60% O_2_) below the ice between two wooden bars placed through core holes and connected by a rope with marks every metre. The method was repeated three times along 30 to 35-m profiles covering a mix of bare ice, and various melt ponds. The spectral energy fluxes between 400 and 700 nm were converted to photon fluxes and integrated to get values for photosynthetically active radiation (PAR).

By identifying measurements made more than about 2 m from pond edges, typical transmittance values for PAR were determined for the various surface types (bare white ice, dark ponds and bright, blue ponds) as in Hudson et al. [1]. The investigated ice floe was then classified into these three surface types (and open water) using thresholds in the red and blue channels, and the resulting surface areas were used to calculate floe-scale transmittance [1].

#### “Inverted sediment trap” approach and relative under-ice current velocities

We collected all aggregates accumulating in a 3.2 m^2^ hole cut through the ice at 12 h intervals from 29 to 1 August to obtain an estimate of aggregate accumulation over time in the surface meltwater layer. We likely underestimated the amount of aggregates as not all of them would have floated up into the ice hole. The aggregate material from each sampling interval was dried at 60°C and the dry weight determined. Triplicate subsamples of 0.5-2 µg dry weight were analyzed for POC and PON. Triplicate measurements had a precision better than 7% for both POC and PON.

Relative current velocities (relative to the drifting ice floe) were measured below the ice with an ice-tethered acoustic doppler current profiler (Nortek 600 kHz Aquadopp) in the vicinity of the ice hole. The shallowest data cell was located about 1.5 m below the ice. These data (means) were extrapolated to 0.2 m below the under-ice surface through a log decay formulation (law-of-the wall approximation [33]). Mean relative current velocities for each sampling interval were then used to extrapolate the accumulated aggregate biomass to the area sampled by our “inverted sediment trap”. The aggregate flux was calculated by correcting the accumulated aggregate biomass for the time between each sampling interval.

### IceArc

#### Sample collection

Aggregates were collected together with ambient seawater using a plastic spoon or bucket. This aggregate-seawater mixture was sub-sampled for POC, PON, pigments and net primary production (NPP) analysis. In order to estimate the dilution factor with ambient seawater, individual aggregates of known volume (10-25 ml) were filtered through pre-combusted GF/F filters for POC and PON analyses as stated above. For pigment analysis including Chl *a*, 1-40 mL of aggregate-seawater mixture, 1 L of melted sea ice, 1 L of seawater and 0.5 L of melt pond water was sampled at stations Ice1 and Ice2 respectively. The ice core was cut into 10 cm sections and each section melted for 24 h in 2 L of 0.2 µm filtered seawater. The samples were filtered on GF/F filters and immediately frozen in liquid nitrogen. Sample storage prior to analysis was at -80°C. Pigments were measured with high-performance liquid chromatography as described in Taylor et al. [34].

For NPP experiments, ice-algal aggregates were collected from the same aggregate-seawater mixture sampled for POC, PON and pigment analysis; seawater was collected 0–2 m below the ice with a peristaltic pump while water from closed melt ponds (without connection to the seawater below) was sampled with a hand pump; ice cores were taken as stated above and divided in two sections: top and bottom. The aggregate mixture, seawater and melt pond samples were directly transferred into incubation bottles (cell culture bottles, Corning Inc., Corning, NY, USA). The ice core sections were melted in the dark for 24 h at 4°C prior to incubation. No filtered seawater was added during melting to avoid addition of nutrients.

Chl *a* concentrations and NPP of the aggregate-seawater mixture were corrected for dilution by a factor calculated from the POC measurements performed in the aggregate-seawater mixture and in individual aggregates in order to derive aggregate specific Chl *a* concentrations and NPP rates.

All samples were collected within 500 m of each other at both ice stations. Water and sea ice samples were taken on the same day while melt pond samples were obtained on the subsequent day but always within 24 h of the other samples.

### ROV transect analysis

Detection of aggregates and their spatial distribution was monitored with an upward-looking video system (Osprey, Tritech, Aberdeen, UK) mounted onto a remotely operated vehicle (V8Sii ROV, Ocean Modules, Åtvidaberg, Sweden) navigated directly under sea ice at stations Ice1 and Ice2 [35].

Aggregates were automatically identified on the video images using a threshold algorithm. Images were extracted each 5 sec from the dive videos. All images were registered according to the distance between camera and ice given by the acoustic altimeter value and cropped leaving the central part of the image in order to remove overlays and edge effects. Images with an ROV tilt of more than 10° and those recorded deeper than 5 m were discarded. Only the green channel of the RGB image was used for analysis, as it showed the clearest aggregate signal. Pixels with a green value <100 (dark pixels, range 0 to 255) were considered as algae. Detection was verified manually. To average over the spatially unevenly distributed data (some locations were photographed several times), all records were grouped in 3×3 m cells. Means were calculated for each cell. Analysis showed that different cell size choices did not significantly change calculated numbers with the exception of total covered area. ROV video material at station Ice1 consisted of four dives covering a total area of 5184 m^2^ and 3 h 52 min of dive video, while at station Ice2 five dives covered a total area of 1809 m^2^ and 3 h 51 min of dive video. Due to ROV-attitude or wrong detection, 34% and 35% of the data from stations Ice1 from station Ice2, respectively, had to be discarded. For details on ROV-dive statistics and up-scaling calculations see Tables S1 and S2 respectively.

### Net primary production

During the IceArc cruise NPP was measured using the ^14^C uptake method [36] with minor modifications. All samples were spiked with 1µCi mL^-1^ of ^14^C sodium bicarbonate. At the end of each incubation period, samples were filtered onto 0.2 µm nitrocellulose filters and the particulate radioactive carbon uptake was determined by liquid scintillation counting using Filter count (PerkinElmer) scintillation cocktail. The average of the dark values was subtracted from the light triplicates. Dissolved inorganic carbon (DIC) was estimated for each sample using the flow injection system [37] and the DIC concentration was taken into account to calculate the amount of labeled bicarbonate incorporated into the cell.

Aggregates were incubated at typical under-ice irradiances, while seawater, sea ice and melt pond samples were incubated at a range of irradiances to calculate the depth-integrated NPP. Aggregates, diluted in ambient water, were mixed well and distributed in 6 cell culture plastic bottles of 10 mL each. One set of triplicates was incubated at 50 µmol photons m^-2^ s^-1^ and the other in the dark at -1.3°C for 24 h (long enough incubation to measure net and not gross primary production). Triplicate measurements for the aggregates had a precision of 7 and 17 % for stations Ice1 and Ice2, respectively. For direct comparison with depth-integrated seawater, melt pond and sea ice NPP, the aggregate NPP was normalized to the dilution-corrected Chl *a* concentration (see sample collection) and up-scaled based on the ROV surveys (Table S3).

Seawater, sea ice and melt pond samples were distributed in 10 cell culture plastic bottles of 20 mL each. Subsequently, they were incubated for 12 h at -1.3°C under a range of light irradiances (0–420 µmol photons m^-2^ s^-1^). *In situ* NPP rates were inferred for each metre of water depth and for each 10 cm of sea ice and melt pond depth from the Chl *a* normalized photosynthesis-irradiance curves (P-E curves) (r^2^ = 0.83-0.96) [38], as a function of the PAR available at each depth. These values were calculated from the daily average incoming PAR, measured with a pyranometer mounted on the ship, and the light attenuation coefficients of 1.5 m^-1^ for sea ice [39] and 0.1 m^-1^ for Atlantic-influenced Arctic seawater, based on data from the TransArc expedition ARK-XXVI/3 to the same area during the previous year. Subsequently, these Chl *a*-normalized rates were multiplied by the measured Chl *a* profile and summed up to give the depth-integrated NPP rates. For the water column, NPP was integrated over the euphotic zone (1% light depth).

Sea ice observations and station data from the IceArc cruise are freely available in PANGAEA, the Data Publisher for Earth & Environmental Science (http://doi.pangaea.de/10.1594/PANGAEA.803221 and http://doi.pangaea.de/10.1594/PANGAEA.803115) while aggregate NPP, TEP and species composition, “inverted sediment trap” calculations and the oxygen profiles are archived in the Marine data-base of the Norwegian Polar Institute and publicly available under http://data.npolar.no/dataset/67eede8a-fe8f-11e2-ba11-005056ad0004.

## Results

### ICE12 cruise

**Figure 2:**
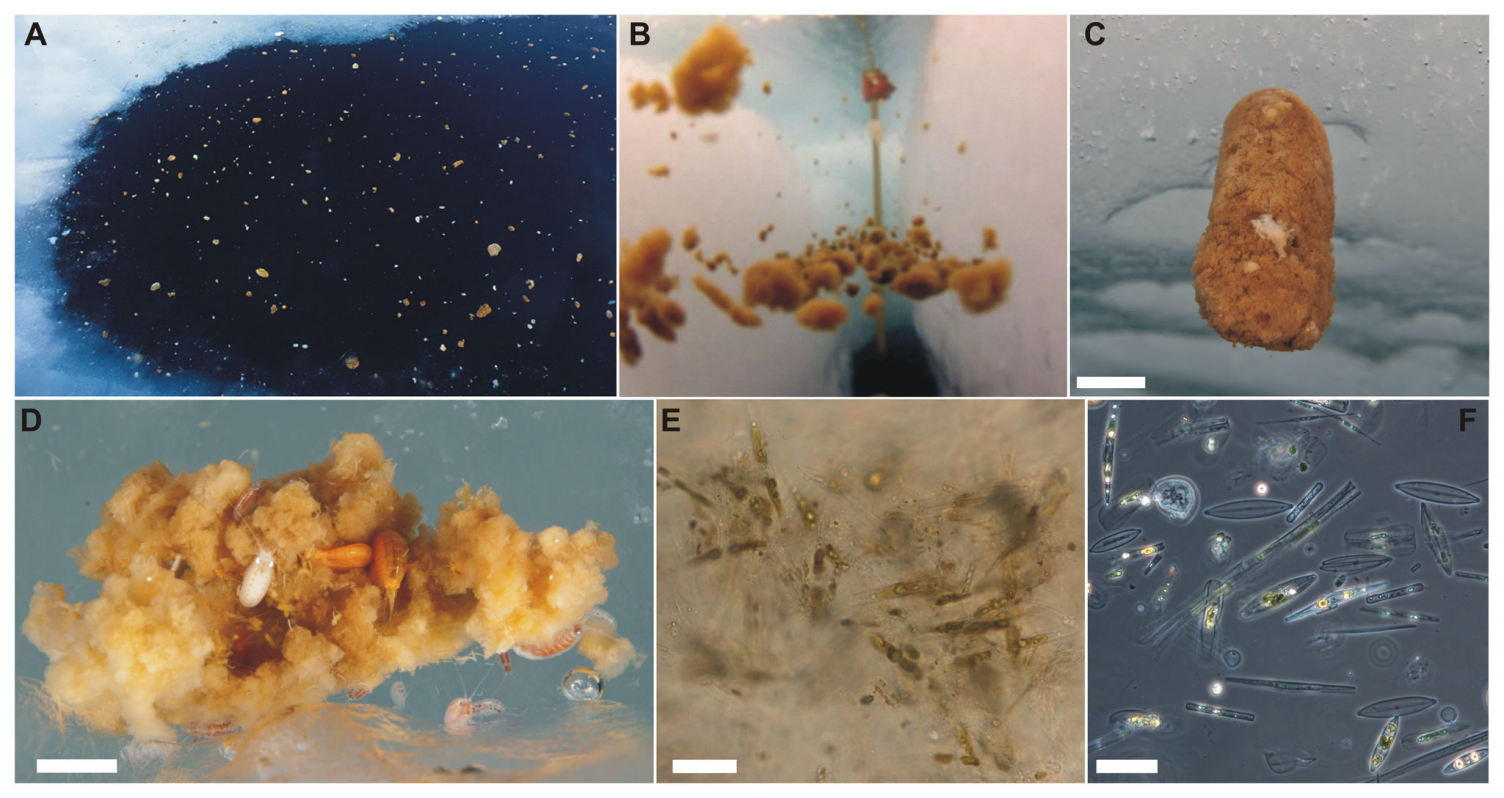
Distribution and composition of ice-algal aggregates. (A) Mass accumulation of aggregates in open melt pond. (B) Accumulation of aggregates at the interface between melt and seawater layers in a natural crack through the ice. (C) Composite aggregate floating beneath sea ice. (D) Porous aggregate colonized by the ice amphipod species *Apherusa glacialis* (white) and *Onisimus glacialis* (yellow). (E) Light micrograph of pennate diatoms, mainly *Hantzschia weyprechtii*, embedded in the mucous matrix. (F) Light micrograph of mainly empty frustules of different pennate diatom species. Scale bar = 0.5 m(A), 5 cm (C), 1 cm (D) and 20 µm (E and F). In panel B the measuring bar (1 m) lowered into the ice crack in the background serves as a scale for orientation. Picture A © Jenny E. Ross, pictures C and D © Peter Leopold.

The investigated ice floe consisted of FYI and covered a total area of 0.5 km^2^. The ice was characterized by a modal thickness of 0.8 m measured using an airborne electromagnetic induction device (the “EM-bird”) towed above the ice by helicopter [40] and a 23% melt pond fraction based on aerial photography [1]. The average PAR transmittance of white ice, bright ponds and dark ponds was 0.17, 0.32, and 0.61, respectively. For the investigated ice floe, the calculated average transmittance for PAR was 0.25. On the three days that radiation transects were made (all within an hour of local noon under cloud cover), the incident PAR averaged 533 µmol m^-2^ s^-1^, with a standard deviation and range of 152 µmol m^-2^ s^-1^ and 288-857 µmol m^-2^ s^-1^, respectively. Melting was evidenced by actual thinning of the sea ice measured during the occupation of the drift ice station [1]. A roughly 0.5 m thick freshwater layer was separated from the underlying seawater by a sharp density gradient detected in cracks and openings in the ice at approximately the ice-water interface. Meltwater was also observed by scuba divers to accumulate in domes and pock holes on the ice bottom. Throughout the occupation of the drift ice station, we observed macroscopic aggregates floating within the meltwater layer beneath sea ice and accumulating in under-ice domes, open melt ponds (Figure 2A) and leads. The majority maintained buoyancy just above the sharp pycnocline that separated meltwater and seawater layers (Figure 2B).

The aggregates were relatively compact, had a mucous consistency and were light to dark brown in colour (Figure 2B-D). Whitish aggregates were frequently observed drifting at the surface of open melt ponds or settled onto the melt pond bottom. The aggregates were dominated by typical ice-associated pennate diatoms (Figure 2E, F), in particular *Navicula pelagica*, *Hantzschia weyprechtii*, *Entomoneis paludosa* and *Cylindrotheca closterium* (Table S4). The aggregates were densely packed with cells as illustrated by abundances of >2×10^5^ cells mL^-1^ in case of *N. pelagica*. Centric diatoms were represented by only two species, the epiphytic *Attheya septentrionalis* and *Thalassiosira bioculata* (Table S4). Cells were embedded in a mucous matrix (Figure 2E) and a large fraction of embedded diatoms consisted of empty frustules (Figure 2F). Non-diatom protists were numerically dominated by flagellates and contributed 6-24% of the total protist abundance (Table S4). High abundances of naked ciliates were observed in the aggregates collected on 26 July (Table S4). The species could not be identified with certainty, but were possibly represented by holotrichous ciliates typically found associated with sea ice [19]. These ciliates were actively feeding within the mucous matrix of the aggregates. Larger grazers included ice amphipods, in particular *Apherusa glacialis* and *Onisimus glacialis*, feeding at the surface of the aggregates (Figure 2D). Iodide concentrations in aggregate filtrate were 2.5-fold elevated compared to ambient seawater (Figure S1), while Chl *a* concentrations of 15 ±1 mg per liter of aggregate were roughly five orders of magnitude higher than in the bottom 20 cm of sea ice (1.35 ±1.08 µg L^-1^), the under-ice water column (0.22 ±0.11 µg L^-1^) and melt ponds that had not melted through (0.05 µg L^-1^). Assuming a thickness of 0.5 m for the meltwater layer (a square meter would thus correspond to 500 liter), that the majority of aggregates floated within this layer and were similar in volume to the one measured (38 mL), the Chl *a* concentration of aggregates in a random liter of seawater corrected for their square metre abundances (Table 1) would amount to 0.93 µg L^-1^ at station Ice1, 5.97 µg L^-1^ at station Ice 2 and 0.28 µg L^-1^ during ICE12. These estimates are in the range of those measured for the sea ice and under-ice water column.

**Table 1:** Aggregate parameters derived from ROV-dives and POC and PON measurements at the scale of the individual aggregate (mg L^-1^) and up-scaled to area (mg m^-2^) at stations Ice1 and Ice2.

	Mean	Median						
	Abundance	Diameter	POC		PON		Chl *a*	
Station	(Agg. m^-2^)	(cm)	(mg C L^-1^)	(mg C m^-2^)	(mg N L^-1^)	(mg N m^-2^)	(mg L^-1^)	(mg m^-2^)
Ice1	0.79	1.04	399	0.19	56	0.03	3.67	0.0017
Ice2	5.06	0.87	873	1.33	94	0.17	4.16	0.0063
ICE12	0.24	–	–	0.74	–	0.10	15	–

Aggregate abundance and POC and PON stocks estimated with the “inverted sediment trap” approach during cruise ICE12. See Tables S1 and S2 for details on the ROV-statistics and up-scaling calculations.

TEP concentrations of the two aggregates were 4102 ±205 and 6801 ±997 µg Xeq. L^-1^. In the case of the first aggregate, TEP concentrations normalized to aggregate carbon amounted to 0.241 ± 4.7 x10^-5^ µg Xeq. µg^-1^ C, which corresponds well to earlier observations of aggregates of miscellaneous origin [41]. When converted to carbon, TEP accounted for 15% of bulk aggregate carbon. The aggregate TEP-C:POC ratio lies within the range 10-20% reported previously for seawater of the North Atlantic [30].

**Figure 3:**
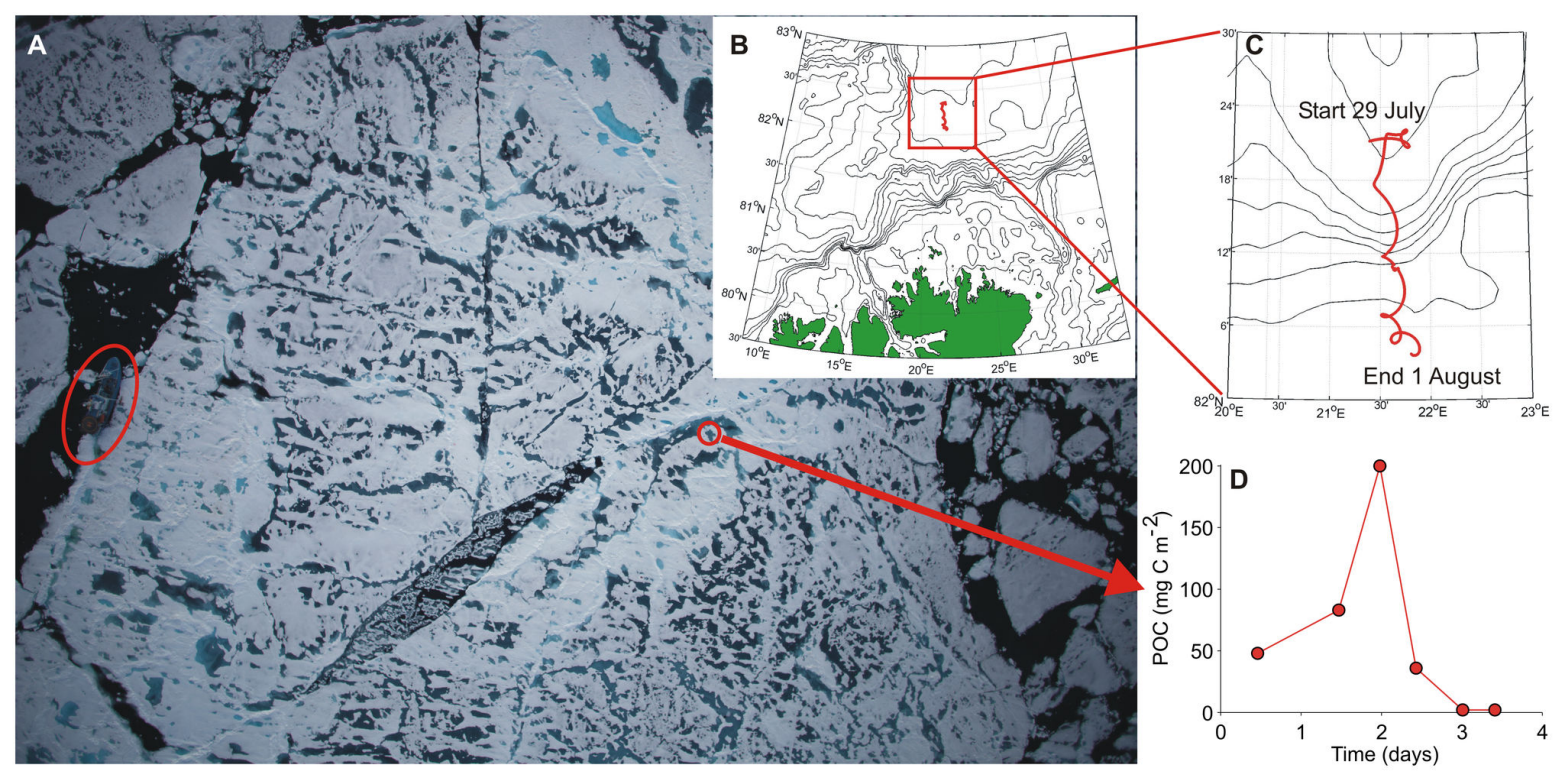
Aerial photograph (A) of the investigated ice floe during ICE12. Map (B) and close-up (C) of the 64 km ice floe drift trajectory north of Svalbard. “Inverted sediment trap” ice-algal aggregate POC time series covering the sampling period from 29 July-1 August (D). The red oval highlights RV *Lance* while the red circle indicates the approximate location of the ice hole. The length of RV *Lance* (60.8 m) can be used as a scale.

During the six sampling intervals between 29 July and 1 August, the ice floe drifted 64 km with a largely southward component and a mean velocity of 0.22 ±0.06 m s^-1^ (Figure 3A-C). Mean under-ice current velocities relative to the drifting ice ranged between 0.08 and 0.19 cm s^-1^ during the sampling period and showed a slight increase with depth. Mean relative current velocity showed a linear relationship with accumulated POC, except for the highest POC value (Figure S2). The cumulative aggregate biomass over the entire sampling period amounted to 0.4 g C m^-2^, while aggregate biomass for each sampling interval increased from initially 48 mg C m^-2^ to 200 mg C m^-2^ by 31 July and declined thereafter (Figure 3D). The aggregate flux increased from initially 104 mg C m^-2^ d^-1^ to 393 mg C m^-2^ d^-1^ by 31 July and declined thereafter to values as low as 3 mg C m^-2^ d^-1^ by 1 August. The mean (±SD) molar POC:PON ratio of the aggregates was 9.7 ±0.7. Corrected for mean relative under-ice current velocities calculated for each sampling interval, we estimated an aggregate standing stock of 0.74 mg C m^-2^ and abundance of 0.24 aggregates m^-2^ over a 499 m^2^ catchment area.

In an adjacent but smaller ice hole, exceptionally high ammonium concentrations of 2.5 µmol L^-1^ were measured during the peak aggregate accumulation on 31 July. Oxygen profiles measured through the same ice hole showed a decrease in oxygen concentrations in the upper 3 m of the under-ice water column between the two sampling occasions on 30 and 31 July 2012 (Figure S3).

### IceArc cruise

Sea ice covered 80% of the ocean surface and was dominated by FYI with a thickness of 1.0-1.2 m and a melt pond coverage of 40% at station Ice1, and a thickness of 1.2-2.0 m and a melt pond coverage of 20% at station Ice2 [42]. Different types of algal aggregates were observed at the eight ice stations occupied during the IceArc cruise. A diverse community of pennate diatoms dominated the aggregates at stations Ice1 and Ice2, while *Melosira arctica*-dominated algal aggregates were found at the remaining ice stations [42]. We will only discuss the former aggregates as the latter have been presented elsewhere [42]. The aggregates collected at stations Ice1 and Ice2 were very similar in shape, texture and species composition (dominance of pennate diatoms) to those recorded during the ICE12 cruise. Aggregate-specific Chl *a* concentrations were roughly four-times lower as compared to ICE12 and aggregate POC and PON concentrations at station Ice2 roughly twice as high as those measured at station Ice1 (Table 1). The almost-exclusive occurrence of the marker pigments fucoxanthin and chlorophyll c1*+2* at station Ice1 (Figure S4C) supports the dominance of diatoms. At Ice2, the occurrence of 19-hexanoyl-oxy-fucoxanthin, chlorophyll *c* 3 as well as chlorophyll b and prasinoxanthin indicated the additional contribution of haptophytes and prasinophytes to the aggregate biomass (Figure S4D). The aggregates from Ice1 showed no chlorophyll degradation products (Figure S4A) indicating a healthy diatom population, which is further reflected in the relatively low POC: Chl *a* ratio of 109 and the near Redfield POC:PON ratio of 7.1. At Ice2, phaeophorbide *a* and pyrophaeophobide *a* indicated grazing, and the relative high proportion of phaeophythin *a* and high POC: Chl a (210) and POC:PON (9.3) ratios further suggested the occurrence of senescent algae in the aggregates (Figure S4B).

**Figure 4:**
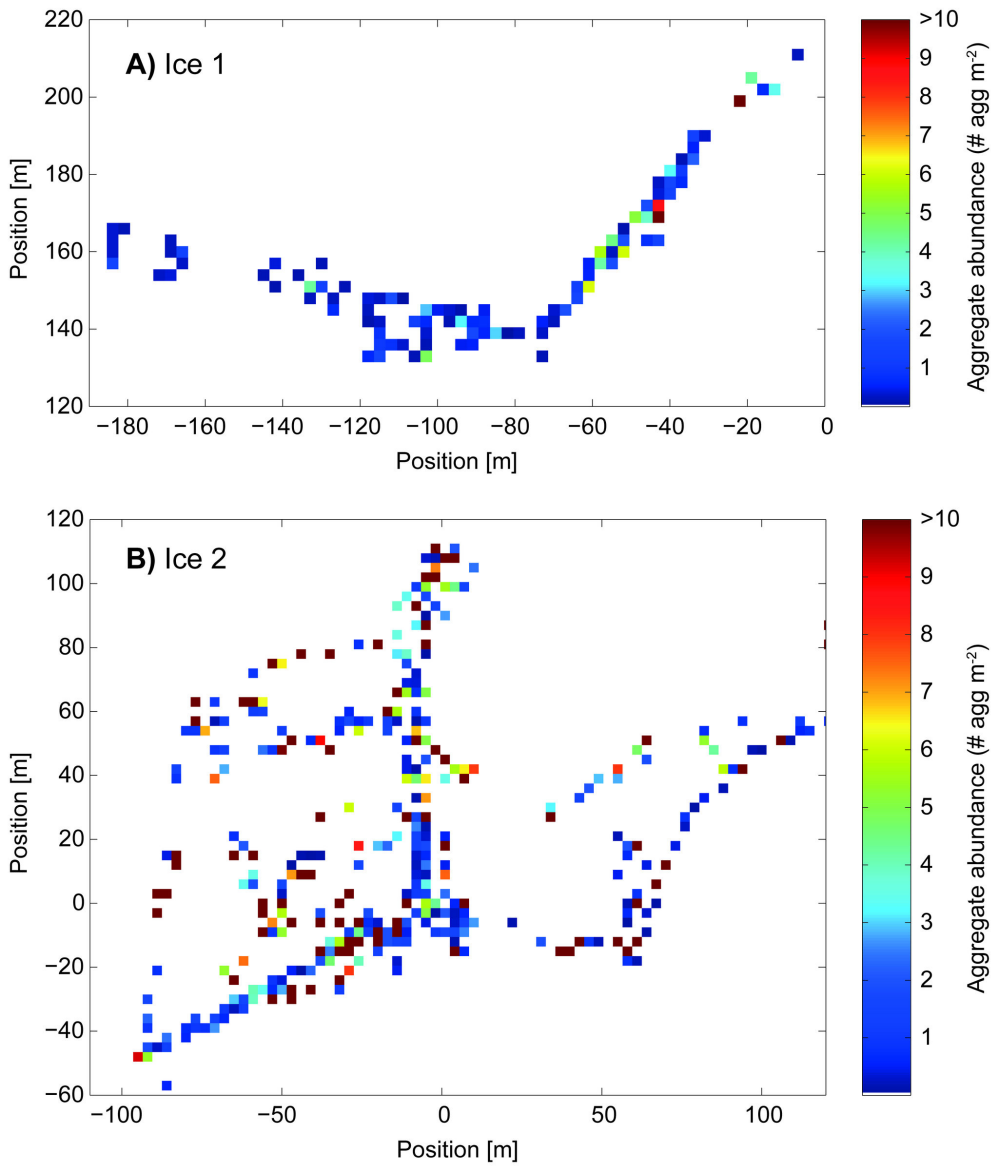
Abundance distribution of ice-algal aggregates at stations (A) Ice1 and (B) Ice2 measured with an upward looking camera mounted onto a remotely operated vehicle. The positions are coordinates (in m) within a floe fixed coordinate system, which was established on both stations.

Ice-algal aggregates exhibited a patchy distribution underneath the sea ice, with maximum abundances of >10 aggregates m^-2^ (Figure 4). Mean aggregate abundances at stations Ice1 and Ice2 (Table 1) were higher than those recorded during the ICE12 cruise. The aggregates covered on average 0.01 and 0.03% of the area sampled during the ROV surveys at stations Ice1 and Ice2 respectively. The vast majority of aggregates ranged from <1 cm to 15 cm in diameter and those between 3 and 12 cm in diameter accounted for 80 to 90% of the cumulative aggregate volume. Aggregates >15 cm were occasionally observed, but due to the limited number of observations were not included in the statistical analysis. Furthermore, the assumption of a spherical shape did not apply to these large aggregates as they were more likely to represent patches of individual aggregates closely aligned or stuck to each other.

Aggregate NPP rates per volume exceeded those measured in melt ponds, sea ice and the water column by 2-4 orders of magnitude (Table 2). ROV-derived aggregate abundances and areal percentage of aggregate cover allowed us to upscale our measurements of individual aggregates to a square-metre basis. Carbon standing stocks differed by almost one order of magnitude between station Ice1 (0.19 mg C m^-2^) and Ice2 (1.33 mg C m^-2^) (Table 1). The mean value derived from the “inverted sediment trap” approach during ICE12 (0.74 mg C m^-2^) falls in between these two estimates (Table 1). Areal aggregate NPP at station Ice2 was an order of magnitude higher than at station Ice1 and in the same range as depth-integrated melt pond NPP (Table 2). However, the remaining depth-integrated melt pond, sea ice and the under-ice water column NPP rates were 2-3 orders of magnitude higher than those measured for the aggregates (Table 2).

**Table 2:** Net primary productivity (NPP) at the scale of the individual aggregate (mg C m^-3^ d^-1^) and up-scaled to a square-metre area (mg C m^-2^ d^-1^).

	NPP		Up-scaling NPP	
	(mg C m^-3^ d^-1^)		(mg C m^-2^ d^-1^)	
	Ice1	Ice2	Ice1	Ice2
Aggregate	3636	10304	0.002	0.02
Melt pond	8.6	0.2	8.4	0.04
Sea ice	4.4	2.0	9.2	1.0
Water column	2.1	1.1	19.4	2.0

Depth-integrated water column NPP was calculated for the euphotic zone (1% light depth). Depth-integrated melt pond and sea ice NPP was calculated for the entire pond depth and ice thickness, respectively. See Table S3 for details on the NPP up-scaling calculations.

## Discussion

### Formation, source and properties of ice-algal aggregates

The FYI encountered during this study was in a late stage of melt as indicated by a low modal ice thickness, high PAR transmittance, well-developed melt ponds and a freshwater layer below the ice [1]. Chl *a* concentrations were low both in sea ice and the underlying water column. As we did not observe the formation of the floating ice-algal aggregates described herein, the conditions conducive to their formation had already been established prior to our investigation period. Interestingly, all previous observations [18-21] were made during the melt season, which in itself indicates that the aggregates originated from the melting sea ice. Strong supportive evidence of this is provided by the observed species composition of the aggregates. Indeed, ice-associated diatoms dominated within the aggregates while pelagic species were conspicuously rare. We cannot rule out that the abundance of flagellates and naked ciliates within the aggregates was underestimated due to the aldehyde mixture used; however, we chose this preservation method precisely because in previous studies it resulted in no significant loss of fragile flagellates [31,43]. The holotrichous ciliate species living within the aggregate matrix were rare in the water column, implying that the aggregate matrix provided an optimal food supply and possibly some protection from pelagic grazers. These ciliates are usually predominantly bacterivorous, and were probably feeding on bacteria fuelled by dissolved organic carbon and TEP released from senescent diatoms [44].

The species composition clearly distinguished our aggregates from the metre-long filaments or strand-like aggregates formed by the centric sea ice diatom *Melosira arctica* [17,42,45-47] and those formed after phytoplankton blooms [48]. The ice-algal aggregates and those formed by *M. arctica* have been summarized under sub-ice assemblages [49] and further categorized by Melnikov [20] as plankto-benthic and benthic types respectively. Strand-like aggregates formed predominantly by *M. arctica* are attached to the underside of the ice, but grow in the underlying water column. The floating ice-algal aggregates we observed originated from the interstitial assemblage dominated by pennate diatoms that grow in the bottom of sea ice and are embedded in a mucous matrix, reminiscent of a biofilm. This continuous, inter-connected community likely already sloughed off from the bottom of the sea ice during the initial stages of melting as the darker ice-algal patches on the underside of sea ice accelerate bottom ablation. Extensive flushing of sea ice through melt pond drainage and higher light availability under thinning ice [1,50-52] likely further facilitated the formation of the floating ice-algal aggregates.

Extracellular polymeric substances (EPS) have been proposed as a binding agent of aggregates [53] and an adaptation employed by sea ice diatoms to survive the cold and saline conditions characteristic of sea ice brine channels [54]. Indeed, EPS made up >68% of dissolved carbohydrates in different sea ice habitats encountered in the Weddell Sea in 2005 and 2006 [55]. Some researchers have addressed the potential buoyancy of microorganisms upon release from melting sea ice as a result of EPS produced by ice algae [56]. Ice-algal EPS may even contribute to the release of ice-algae into the under-ice water column, as it has been suggested that EPS alter the melting rate of Arctic sea ice [57]. Interestingly, a considerable fraction of the EPS network seems to remain attached to the ice bottom even after the loss of the algae and could explain the carbon pools found in sea ice after the termination of the ice-algal bloom [58]. TEPs constitute a special type of sticky mucopolysaccharide gels, a subcategory of EPS [30] that are formed from dissolved precursors released from actively-growing or senescent phytoplankton and facilitate the aggregation of solid, non-sticky particles [59]. In the Arctic, it has been shown that the majority of TEP underneath first-year summer pack ice is produced by diatoms [60]. Once algal material is dislodged from melting sea ice, the sticky nature of TEP and collision of individual particles, when they collect in domes and crevices in the ice, will favour coagulation of the free-floating algal material into larger composite aggregates.

### Distribution and ecological significance

The distribution of ice-algal aggregates underneath sea ice was very patchy as evidenced by the large temporal variability in accumulated aggregate biomass inside our ice hole, and during diver observations and spatial ROV surveys. The skewed size-frequency distribution illustrates that the majority of ROV-detected aggregates were smaller <15 cm and likely represent individual aggregates. This is supported by the ICE12 drift station observations. However, occasional detections of patches >15 cm represent accumulations of individual aggregates that concentrate in under-ice domes, open melt ponds or leads as observed by divers and when surface sampling during ICE12. The lower aggregate abundance estimated with the “inverted sediment trap” approach, as compared to the ROV surveys, indicates that aggregates are easily transported below the ice before they settle in domes or crevices. This was also observed by divers, since exhaled air or fin movements rapidly dislodged and often dismantled the floating aggregates. Our “inverted sediment trap” estimate of aggregate abundance is therefore conservative because not every passing aggregate got trapped. Nevertheless, it lies in the range reported from an earlier study based on dive transects [20]. Changes in current speed and direction also influenced the sampling by our “inverted sediment trap”, as reflected in the positive, linear relationship between mean relative current velocity and aggregate flux. The fact that this relationship did not apply for the highest accumulated aggregate biomass on 31 July was likely due to the very patchy distribution of the aggregates or changes in current direction. On that day, our “inverted sediment trap” might have sampled an area with exceptionally high aggregate abundance. Despite lower abundances overall, aggregate standing stock extrapolated to the catchment area of our “inverted sediment trap” lies within the range calculated from the ROV surveys. This is because the aggregates were generally larger than those measured during the ROV transects. In addition to the differences in methodology, comparisons are further complicated by differences in spatial coverage and duration between the “inverted sediment trap” approach and the ROV surveys. The ROV surveys covered, within a few hours, a three to 10-fold larger area as compared to the area sampled for 3.5 days by the “inverted sediment trap”. Given the characteristically patchy nature of sea-ice habitats, many of the differences (or similarities) in the data may therefore simply be due to differences in location and/or sampling duration.

Aggregate NPP and standing stocks m^-2^ are small when compared to depth-integrated ice-algal biomass and primary production rates reported for the Arctic, which range from 1-340 mg C m^-2^ in the former and <1-463 mg C m^-2^ d^-1^ in the latter [61]. However, in locations where they accumulate, they constitute a highly concentrated food source for the ice-associated fauna during the oligotrophic summer months, as revealed by high abundances of ice amphipods and ciliates associated with them. Ice amphipods, such as *Apherusa glacialis*, are able to swim in the boundary layer below the ice and adapted to exploit patchy food sources [62]. Furthermore the feeding mode of herbivorous, ice-associated amphipods is not well-suited to efficiently feed on highly diluted and small-sized suspended particles typical of the summer phytoplankton community [63]. Such aggregations could thus constitute an important trophic baseline for specialized sympagic fauna during the melt season, when many organisms, such as ice amphipods, need to rely on degraded material or detritus as a food source [62]. In cases when aggregates are refrozen into the ice during autumn they could also extend food availability into the winter months [17,64] and act as a seeding stock for the next spring. Frozen-in algal aggregates have been observed by divers, particularly during spring (H. Hop, diving obs.). However, this fraction appears to be small compared to the fraction that sinks once the aggregates have lost buoyancy control. This has been shown for *Melosira arctica* dominated aggregates [42,47]. The aggregates found on the sea floor in 3485 m depth at station Ice2 were dominated by pennate diatoms [42] similar to those described herein which indicates that a significant fraction of the floating aggregates eventually lost buoyancy control and sank to the sea floor.

The algal aggregates supported high levels of biological activity on the scale of individual aggregates as revealed by Chl *a* concentrations and NPP several orders of magnitude higher than in the surrounding water column and sea ice. High POC:PON and POC: Chl *a* ratios inside the aggregates during ICE12 and at station Ice2 were likely mediated by preferential bacterial degradation of labile organic matter, in particular PON, and could explain oxygen consumption and elevated ammonium concentrations in surface waters where aggregates accumulated. The occurrence of the Chl *a* degradation products phaeophorbide *a* and pyrophaeophorbide *a* at station Ice2 indicated increased grazing [65]. Older aggregates further seem to accumulate prasinophytes and haptophytes while floating through the under-ice water column because these taxa are usually not prominent in sea ice biota. The high proportion of phaeophythin *a* indicates a high fraction of senescent algae [66] within the aggregates. This is corroborated by the frequent occurrence of bleached aggregates and elevated levels of iodide [27] within the aggregates, indicating that a considerable fraction of embedded algae was either in senescent condition or dead, possibly due to photo-oxidative stress induced by high light levels near the surface. Indeed, the majority of bleached ice-algal aggregates were observed in open melt ponds and sea ice crevices where they were exposed to high levels of incident radiation.

### Historical context

The aggregates studied herein are reminiscent in shape, colour, dominance of pennate diatoms and association with the meltwater layer of the aggregates reported from the Norwegian Fram Expedition in 1894 [18,19] and the Russian North Pole drift ice station NP-23 [20] in 1977 (Figure 1), indicating that the floating ice-algal aggregates described herein are not a new phenomenon. Free-floating algal masses were also observed during the SHEBA (Surface Heat Budget of the Arctic Ocean) ice camp drift in the Canadian Basin, but the aggregations were dominated by two centric diatoms, *Chaetoceros socialis* and *Melosira arctica*, and the epiphytic diatom *Synedropsis hyperborea* [21], representing a different type of algal aggregation. Common to all previous observations is that they were made during drift ice stations that were occupied for at least one full seasonal cycle and thus covered the critical time window during the melt season. Although intensively looked for during a summer cruise to the Arctic Ocean in 2013, we did not observe floating macroscopic aggregates in the same general area and during the same time of the year as the ICE12 drift-ice study. Also during the Fram Expedition, floating ice-algal aggregates were observed in the summer of 1894 but not in the following summer despite intensive efforts to find them [18], suggesting that the occurrence of such events is likely ephemeral and restricted to the melt season.

## Conclusions

Although the biomass associated with the floating ice-algal aggregates was low compared to ice-algal blooms, they sustained high rates of biological activity at the scale of the individual aggregate and provided a concentrated food source for the ice-associated fauna during the oligotrophic Arctic summer months. This type of aggregate-based habitat is likely to be fairly unique because it constitutes an extension of the sea ice community into the under-ice water column during the Arctic summer melt season. The potential significance of the ice-algal aggregates for surface consumption, energy transfer to the benthos and seeding of the next spring bloom remains an interesting topic for future studies, and is an urgent reminder to improve our understanding of the rapidly changing Arctic sea ice ecosystem.

## Supporting Information

Table S1 **Results of aggregate extraction from ROV video transects at stations Ice1 (five dives) and Ice2 (four dives).**
(DOCX)

Table S2 **Up-scaling of aggregate biomass.**
(DOCX)

Table S3 **Up-scaling of Chl *a* normalized aggregate net primary production (NPP).**
(DOCX)

Table S4 **Composition of ice-algal aggregates.**
(DOCX)

Figure S1 **Iodide concentrations measured in aggregate filtrate and ambient surface seawater during cruise ICE12, July-August 2012.** (SD = ±1.8 nM and ±2.4 nM for aggregate sample and surface water samples respectively; n = 3).
(TIF)

Figure S2 **Relationship between mean relative under-ice current velocities (exemplified here for 3.5 m below the ice) and accumulated POC at each sampling interval between 29 July and 1 August, 2012.**
(TIF)

Figure S3 **Oxygen profiles from the upper 5.5 m of the under-ice water column measured with a MicroCat oxygen sensor on 30 and 31 July, 2012.**
(TIF)

Figure S4 **Pigment composition of ice-algal aggregates.** **Marker pigments and chlorophyll and its degradation products at stations Ice1 (A, C) and Ice2 (B, D)**. Chl *c* 3 = chlorophyll *c* 3, Chl *c1+2* = chlorophyll c1*+2*, 19-hex = 19-hexanoyloxyfucoxanthin, Fuco = fucoxanthin, Prasino = prasinoxanthin, Chl *b* = chlorophyll b, Phorbid *a* = phaeophorbide *a*, PyroPhorbid = pyrophaeophorbide *a*, Chl *a* = chlorophyll *a*, Phythin *a* = phaeophythin *a*.
(TIF)
