# Supplementary material for: Floating Ice-Algal Aggregates below Melting Arctic Sea Ice
Source: PLoS One. 2013 Oct 16;8(10):e76599. doi: 10.1371/journal.pone.0076599 (PMC3804104; doi:10.1371/journal.pone.0076599)
Supplement: Table S1 — Results of aggregate extraction from ROV video transects at stations Ice1 (five dives) and Ice2 (four dives). (DOCX) [file pone.0076599.s001.docx]

**Table S1 Results of aggregate extraction from ROV video transects at stations Ice1 (five dives) and Ice2 (four dives).** Aggregate abundance and diameter values used for up-scaling calculations are marked in bold.

| Station | Mean | Median | Max | Min | STD |
| --- | --- | --- | --- | --- | --- |
| Altimeter reading [m]  (Distance between camera and ice-underside) | | | | | |
| Ice1  Ice2 | 1.10  1.04 | 0.98  0.81 | 3.94  5.16 | 0  0 | 0.59  0.90 |
| Areal percentage of algae cover [%] | | | | | |
| Ice1  Ice2 | 0.009  0.034 | 0.0005  0.0020 | 0.23  2.32 | 0  0 | 0.03  0.13 |
| Image resolution (pixel size) [m] | | | | | |
| Ice1  Ice2 | 0.005  0.004 | 0.004  0.003 | 0.018  0.020 | 0  0 | 0.002  0.003 |
| Mean Area covered by images [m²] | | | | | |
| Ice1  Ice2 | 1.60  1.55 | 0.98  0.55 | 16.74  21.50 | 0  0 | 2.05  3.03 |
| Abundance [Agg. m^-^²] | | | | | |
| Ice1  Ice2 | **0.79**  **5.06** | 0.06  0.43 | 17.88  251.3 | 0  0 | 1.99  16.23 |
| Equivalent Aggregate Diameter [m] | | | | | |
| Ice1  Ice2 | 0.016  0.015 | **0.010**  **0.009** | 0.283  0.295 | 0  0 | 0.018  0.019 |
