# Supplementary material for: Floating Ice-Algal Aggregates below Melting Arctic Sea Ice
Source: PLoS One. 2013 Oct 16;8(10):e76599. doi: 10.1371/journal.pone.0076599 (PMC3804104; doi:10.1371/journal.pone.0076599)
Supplement: Table S2 — Up-scaling of aggregate biomass. (DOCX) [file pone.0076599.s002.docx]

**Table S2** **Up-scaling of aggregate biomass.**

Parameters derived from the ROV aggregate detection were used to calculate aggregate volume from the median diameter value, assuming spherical shape. To obtain realistic values we used the median rather than mean diameter values.

Multiplying by mean abundances, we were able to give spatially up-scaled values. Using the mean here accounts for the patchy distribution.

**Measurements:**

| Station | POC  [mg C L^-1^] | PON  [mg N L^-1^] | Chl *a*  [mg L^-1^] |
| --- | --- | --- | --- |
| Ice1 | 399 | 56 | 3.67 |
| Ice2 | 873 | 94 | 4.16 |

**Up-scaling:**

| Station | POC  [mg C m^-2^] | PON  [mg N m^-2^] | Chl *a*  [mg m^-2^] |
| --- | --- | --- | --- |
| Ice1 | 0.19^*^ | 0.0263 | 0.0017 |
| Ice2 | 1.33^¥^ | 0.17 | 0.0063 |

^*^based on:

**POC example for Ice1**:

Median diameter (d): 0.010 m

Mean volume: $\pi\times\frac{d^{3}}{6}=0.0006 L {Agg}^{-1}$

Mean abundance: $0.79 L {Agg m}^{-2}$

$399 mg C L^{-1}\times0.0006 L {Agg}^{-1}\times0.79 Agg m^{-2}=0.19 mg C m^{-2}$

1. 0.34 g C in the survey area (1800 m²)
2. 95 g C in 0.5 km²

^¥^based on:

**POC example for Ice2**:

Median diameter (d): 0.009 m

Mean volume: $\pi\times\frac{d^{3}}{6}=0.0003 L {Agg}^{-1}$

Mean abundance: $5.06 L {Agg m}^{-2}$

$873 mg C L^{-1}\times0.0003 L {Agg}^{-1}\times5.06 Agg m^{-2}=1.33 mg C m^{-2}$

1. 6.65 g C in the survey area (5 000 m²)
2. 665 g C in 0.5 km²
