# Supplementary material for: Floating Ice-Algal Aggregates below Melting Arctic Sea Ice
Source: PLoS One. 2013 Oct 16;8(10):e76599. doi: 10.1371/journal.pone.0076599 (PMC3804104; doi:10.1371/journal.pone.0076599)
Supplement: Table S3 — Up-scaling of Chl a normalized aggregate net primary production (NPP). (DOCX) [file pone.0076599.s003.docx]

**Table S3** **Up-scaling of Chl *a*-normalized aggregate net primary production (NPP).**

The aggregate NPP was divided by the Chl *a* concentration of the aggregate-seawater mixture used for the incubations and then multiplied by the up-scaled Chl *a* standing stocks (see Table S2) in order to derive Chl *a*-normalized aggregate NPP.

**Measurements:**

| Station | NPP  [mg C L^-1^ d^-1^] |
| --- | --- |
| Ice1 | 3.64 |
| Ice2 | 10.3 |

**Chl *a* normalized NPP per square meter:**

| Station | NPP  [mg C m^-^² d^-1^] |
| --- | --- |
| Ice1 | 0.002^*^ |
| Ice2 | 0.02^¥^ |

^*^based on:

**Ice1**:

NPP: 3.64 mg C L^-1^ d^-1^

Chl *a*: 3.67 mg L^-1^

Up-scaled Chl *a* standing stock: 0.0017 mg m^-2^

3.64 mg C L^-1^ d^-1^ / 3.67 mg L^-1^ × 0.0017 mg m^-2^ = 0.002 mg C L^-1^ d^-1^

^¥^based on:

**Ice2**:

NPP: 10.3 mg C L^-1^ d^-1^

Chl *a*: 4.16 mg L^-1^

Up-scaled Chl *a* standing stock: 0.0063 mg m^-2^

10.3 mg C L^-1^ d^-1^ / 4.16 mg L^-1^ × 0.0063 mg m^-2^ = 0.02 mg C L^-1^ d^-1^
