# Supplementary material for: Floating Ice-Algal Aggregates below Melting Arctic Sea Ice
Source: PLoS One. 2013 Oct 16;8(10):e76599. doi: 10.1371/journal.pone.0076599 (PMC3804104; doi:10.1371/journal.pone.0076599)
Supplement: Table S4 — Composition of ice-algal aggregates. (DOCX) [file pone.0076599.s004.docx]

**Table S4** **Composition of ice-algal aggregates.**

Absolute (cells mL^-1^) and relative (%) species composition of aggregates sampled on 26 and 29 July 2012.

|  |  | 26 July | | 29 July | |
| --- | --- | --- | --- | --- | --- |
| Group | Taxon | Cells mL^-1^ | % | Cells mL^-1^ | % |
| Diatoms | *Attheya septentrionalis* | 20487 | 4.14 | 52643 | 9.60 |
|  | *Cylindrotheca closterium* | 37807 | 7.64 | 59839 | 10.91 |
|  | *Entomoneis kjellmanii* | 753 | 0.15 | 3036 | 0.55 |
|  | *Entomoneis paludosa* | 60272 | 12.18 | 35244 | 6.43 |
|  | *Fragilariopsis cylindrus* | 6022 | 1.22 | 13964 | 2.55 |
|  | *Hantzschia weyprechtii* | 62071 | 12.55 | 68177 | 12.43 |
|  | *Navicula directa* | 1505 | 0.30 | 607 | 0.11 |
|  | *Navicula pelagica* | 129962 | 26.27 | 218883 | 39.92 |
|  | *Navicula septentrionalis* | 2635 | 0.53 | 0 | 0.00 |
|  | *Navicula* sp. | 376 | 0.08 | 1821 | 0.33 |
|  | *Navicula transitans* | 2635 | 0.53 | 1214 | 0.22 |
|  | *Navicula trigonocephala* | 3011 | 0.61 | 607 | 0.11 |
|  | *Navicula vanhoeffenii* | 0 | 0.00 | 4857 | 0.89 |
|  | *Nitzschia arctica* | 0 | 0.00 | 607 | 0.11 |
|  | *Nitzschia frigida* | 0 | 0.00 | 4250 | 0.78 |
|  | *Nitzschia laevissima* | 6398 | 1.29 | 8500 | 1.55 |
|  | *Nitzschia promare* | 1129 | 0.23 | 0 | 0.00 |
|  | *Pseudo-nitzschia delicatissima* | 5269 | 1.07 | 9107 | 1.66 |
|  | *Pseudo-nitzschia granii* | 4893 | 0.99 | 5464 | 1.00 |
|  | *Pseudo-nitzschia pseudodelicatissima* | 23901 | 4.83 | 19428 | 3.54 |
|  | *Pseudo-nitzschia seriata* | 0 | 0.00 | 2428 | 0.44 |
|  | *Pseudogomphonema* sp. | 2635 | 0.53 | 4250 | 0.78 |
|  | *Synedropsis hyperborea* | 0 | 0.00 | 3036 | 0.55 |
|  | *Synedropsis* sp. | 1882 | 0.38 | 0 | 0.00 |
|  | *Thalassiosira bioculata* | 1505 | 0.30 | 0 | 0.00 |
| Choanoflagellates | Choanoflagellates | 1882 | 0.38 | 0 | 0.00 |
| Ciliates | holotrichous forms | 9033 | 1.83 | 0 | 0.00 |
| Cyst | round without spines | 753 | 0.15 | 8500 | 1.55 |
| Flagellates | 3-5 µm | 37369 | 7.55 | 1214 | 0.22 |
|  | 5-7 µm | 19525 | 3.95 | 3643 | 0.66 |
|  | 7-10 µm | 0 | 0.00 | 607 | 0.11 |
|  | 10-20 µm | 43450 | 8.78 | 0 | 0.00 |
| Dinoflagellates | *Gymnodinium* 5-10 µm | 376 | 0.08 | 0 | 0.00 |
|  | *Gymnodinium* 10-20 µm | 1505 | 0.30 | 0 | 0.00 |
|  | *Gymnodinium* sp. | 0 | 0.00 | 607 | 0.11 |
|  | *Polariella glacialis* (cysts) | 376 | 0.08 | 1214 | 0.22 |
|  | *Prorocentrum* sp. | 0 | 0.00 | 607 | 0.11 |
| Prymnesiophytes | *Phaeocystis* sp. | 5269 | 1.07 | 13964 | 2.55 |
|  | Coccolithophores | 0 | 0 | 3036 | 0.55 |
