# Supplementary figures and images for: Floating Ice-Algal Aggregates below Melting Arctic Sea Ice
Source: PLoS One. 2013 Oct 16;8(10):e76599. doi: 10.1371/journal.pone.0076599 (PMC3804104; doi:10.1371/journal.pone.0076599)

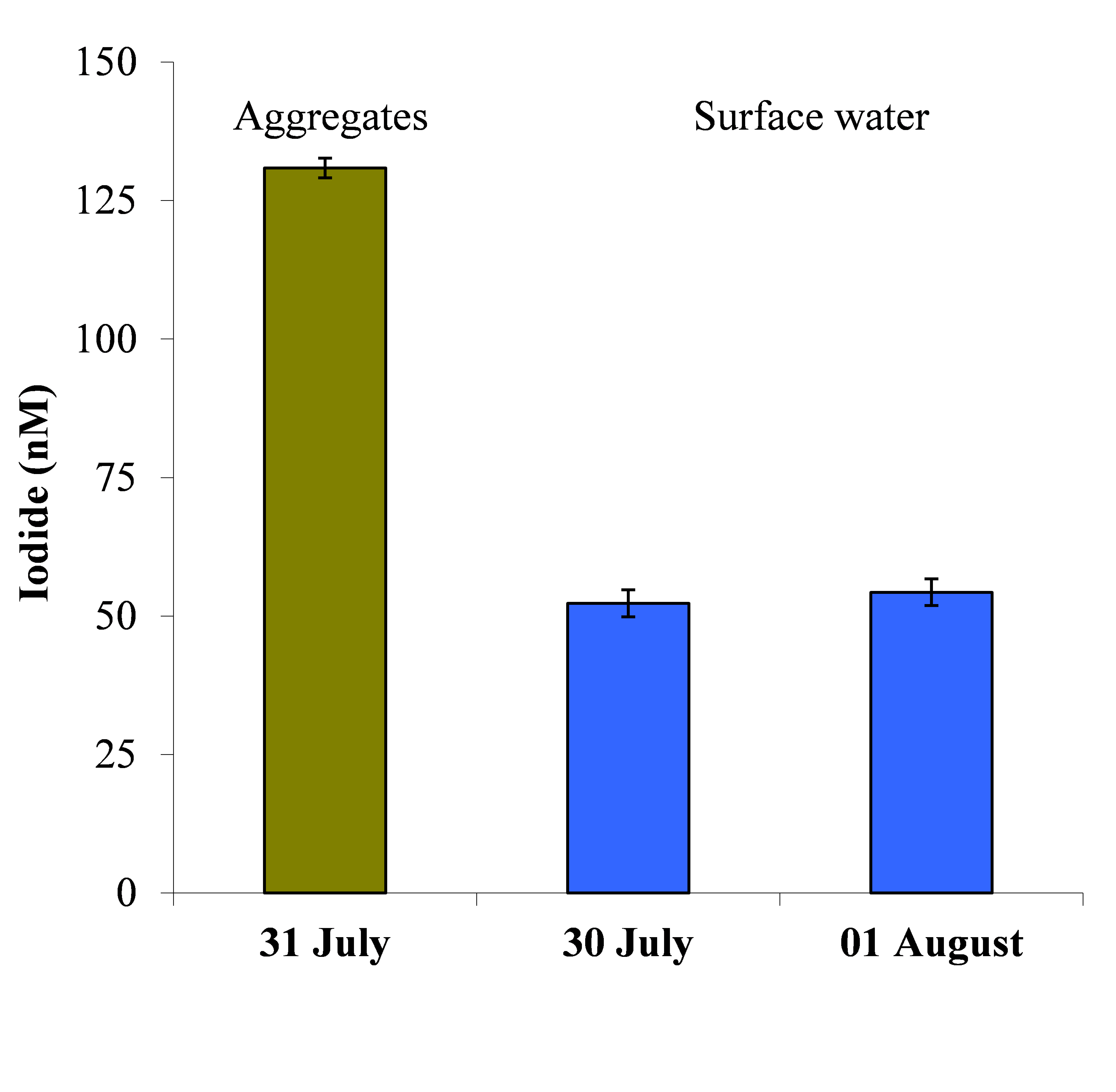

Supplement: Figure S1 — Iodide concentrations measured in aggregate filtrate and ambient surface seawater during cruise ICE12, July-August 2012. (SD = ±1.8 nM and ±2.4 nM for aggregate sample and surface water samples respectively; n = 3). (TIF) [file pone.0076599.s005.tif]

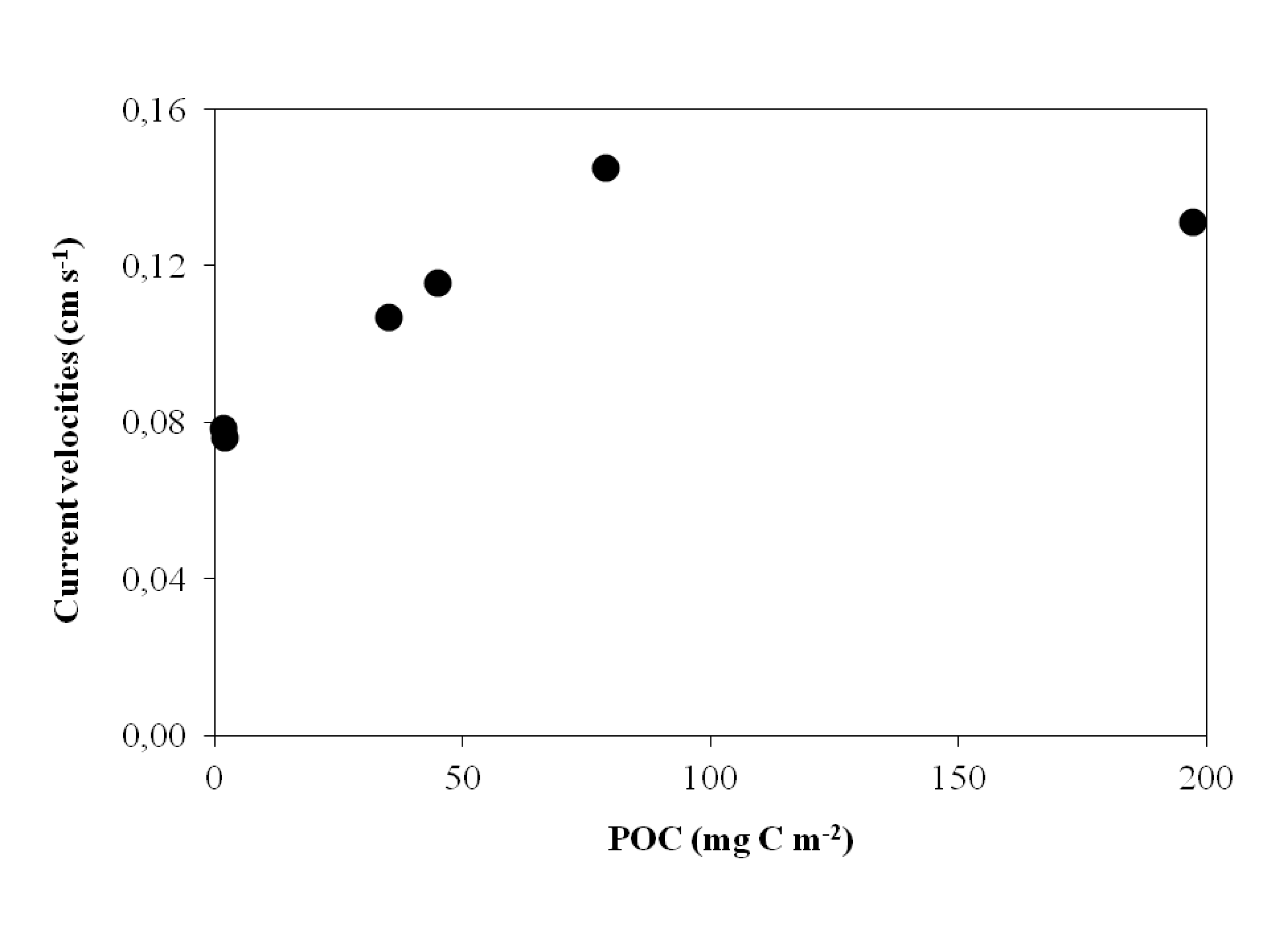

Supplement: Figure S2 — Relationship between mean relative under-ice current velocities (exemplified here for 3.5 m below the ice) and accumulated POC at each sampling interval between 29 July and 1 August, 2012. (TIF) [file pone.0076599.s006.tif]

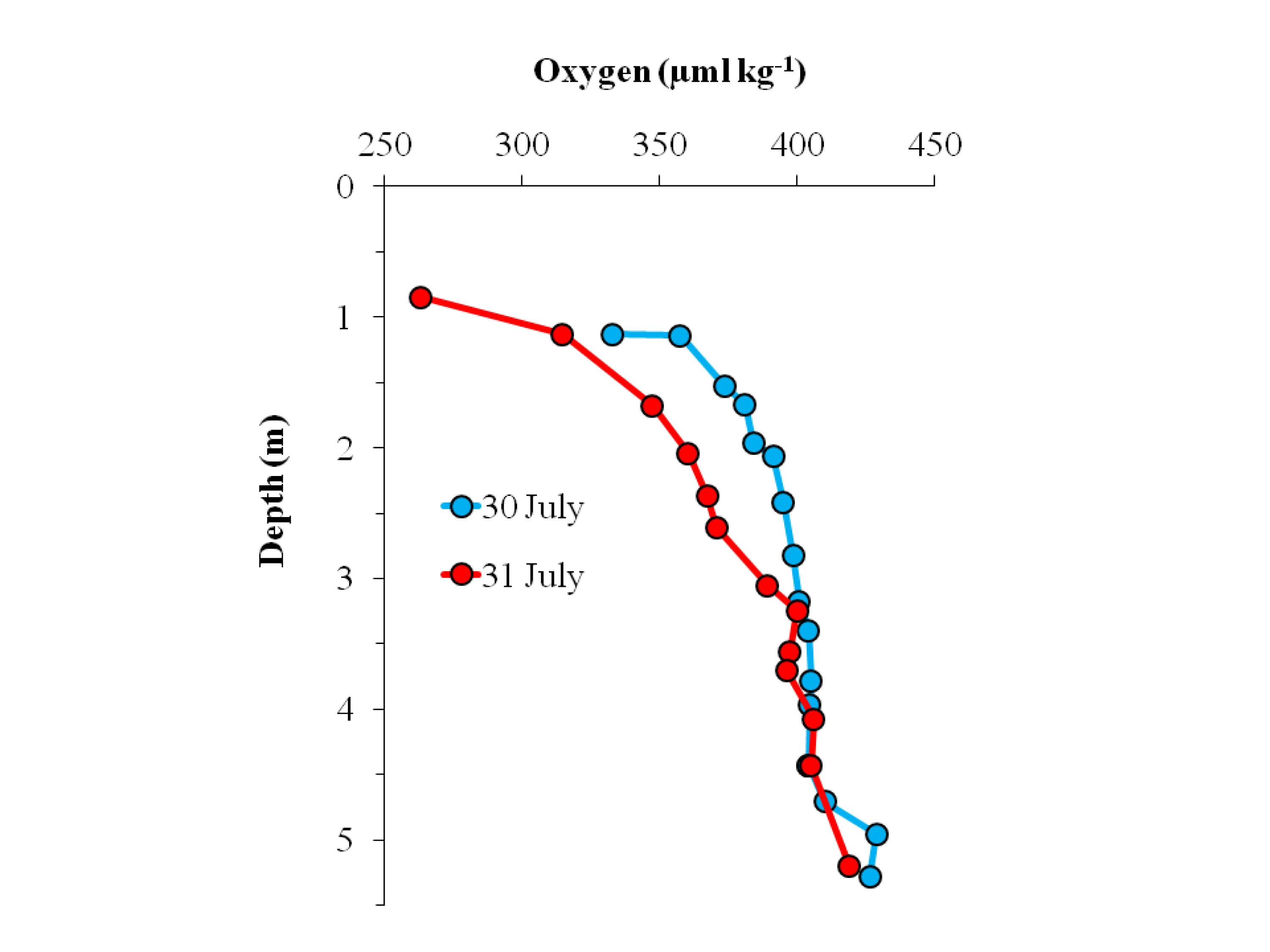

Supplement: Figure S3 — Oxygen profiles from the upper 5.5 m of the under-ice water column measured with a MicroCat oxygen sensor on 30 and 31 July, 2012. (TIF) [file pone.0076599.s007.tif]

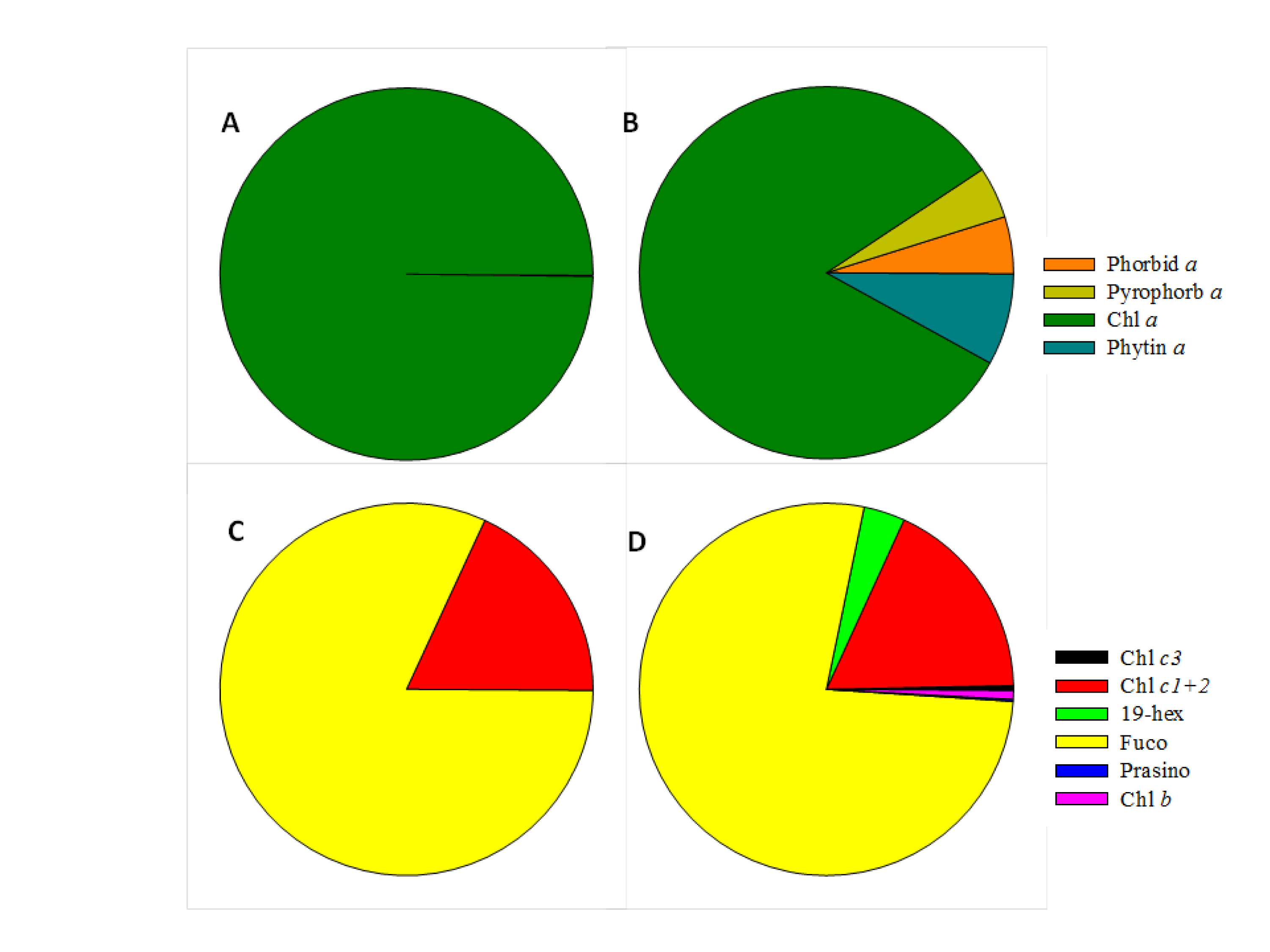

Supplement: Figure S4 — Pigment composition of ice-algal aggregates. Marker pigments and chlorophyll and its degradation products at stations Ice1 (A, C) and Ice2 (B, D). Chl c 3 = chlorophyll c 3, Chl c1+2 = chlorophyll c1+2, 19-hex = 19-hexanoyloxyfucoxanthin, Fuco = fucoxanthin, Prasino = prasinoxanthin, Chl b = chlorophyll b, Phorbid a = phaeophorbide a, PyroPhorbid = pyrophaeophorbide a, Chl a = chlorophyll a, Phythin a = phaeophythin a. (TIF) [file pone.0076599.s008.tif]
